# Cluster analysis of COVID-19 recovery center patients at a clinic in Boston, MA 2021–2022: impact on strategies for access and personalized care

**DOI:** 10.1186/s13690-023-01033-2

**Published:** 2023-03-14

**Authors:** Ann-Marcia C. Tukpah, Jhillika Patel, Beret Amundson, Miguel Linares, Meera Sury, Julie Sullivan, Tajmah Jocelyn, Brenda Kissane, Gerald Weinhouse, Nancy Lange-Vaidya, Daniela Lamas, Khalid Ismail, Chandan Pavuluri, Michael H. Cho, Elizabeth B. Gay, Matthew Moll

**Affiliations:** 1 Division of Pulmonary and Critical Care Medicine, Department of Medicine, Brigham and Women’s Hospital, Boston, MA USA; 2 Robert Wood Johnson Medical School, New Brunswick, NJ USA; 3 Department of Medicine, Brigham and Women’s Hospital, Boston, MA USA; 4 Channing Division of Network Medicine, Brigham and Women’s Hospital, Boston, MA USA

**Keywords:** Informatics, Equity, Disparities, Community health, Quality of care

## Abstract

**Background:**

There are known disparities in COVID-19 resource utilization that may persist during the recovery period for some patients. We sought to define subpopulations of patients seeking COVID-19 recovery care in terms of symptom reporting and care utilization to better personalize their care and to identify ways to improve access to subspecialty care.

**Methods:**

Prospective study of adult patients with prior COVID-19 infection seen in an ambulatory COVID-19 recovery center (CRC) in Boston, Massachusetts from April 2021 to April 2022. Hierarchical clustering with complete linkage to differentiate subpopulations was done with four sociodemographic variables: sex, race, language, and insurance status. Outcomes included ICU admission, utilization of supplementary care, self-report of symptoms.

**Results:**

We included 1285 COVID-19 patients referred to the CRC with a mean age of 47 years, of whom 71% were female and 78% White. We identified 3 unique clusters of patients. Cluster 1 and 3 patients were more likely to have had intensive care unit (ICU) admissions; Cluster 2 were more likely to be White with commercial insurance and a low percentage of ICU admission; Cluster 3 were more likely to be Black/African American or Latino/a and have commercial insurance. Compared to Cluster 2, Cluster 1 patients were more likely to report symptoms (ORs ranging 2.4–3.75) but less likely to use support groups, psychoeducation, or care coordination (all *p* < 0.05). Cluster 3 patients reported greater symptoms with similar levels of community resource utilization.

**Conclusions:**

Within a COVID-19 recovery center, there are distinct groups of patients with different clinical and socio-demographic profiles, which translates to differential resource utilization. These insights from different subpopulations of patients can inform targeted strategies which are tailored to specific patient needs.

**Supplementary Information:**

The online version contains supplementary material available at 10.1186/s13690-023-01033-2.

## Background

COVID-19 disproportionately impacts historically disadvantaged communities of color and patients from socioeconomically under-resourced backgrounds [1]. Individuals with non-White race/ethnicity suffer from more COVID-19 infections and higher COVID-related mortality rates [2–4]. Factors associated with increased risk of COVID infections among racial/ethnic minorities are multifactorial social determinants of health and include underlying comorbidities, occupations that require frequent interactions with the public, housing conditions with limited opportunity for social distancing [2], disparities in access to quality healthcare and historical medical racism resulting in lack of trust in healthcare institutions [5]. These inequities are in part a function of differential resource investment and structural racism resulting in various vulnerabilities [6]. Prior to COVID-19, non-White patients had less reliable access to specialty care [7] and to long-term acute care facilities (mediated by differences in insurance coverage) [8]. These disparities have extended to COVID-19 care. For example, recent evidence suggests that there may be disparities in referral to physical/occupational therapy during an inpatient stay [9], and that living in a neighborhood with greater social vulnerability is associated with organ dysfunction/failure and need for mechanical ventilation [10].

Many post-COVID clinics and multidisciplinary recovery centers have been opened to meet the demand of patients recovering from COVID-19. However, without targeted interventions, post-COVID clinics may perpetuate existing healthcare inequities [11]. Some COVID Recovery clinics have recognized the impact of social determinants of health on recovery and made investments in ensuring equity [12]. Other groups have described recovery processes focused on resilience and rebuilding for communities and health systems, not only for individuals [13, 14]. Indeed, there has been growing awareness of the possibility of disparities in post-COVID care. Despite this raised awareness, little disparities-related quantitative information exists regarding post-COVID symptoms, referrals to recovery centers, and utilization of resources.

We created a COVID Recovery Center (CRC) that not only provides streamlined multi-disciplinary care to post-COVID patients, but also incorporates strategies to address inequalities by facilitating access to socioeconomic status (SES)-targeted interventions. We also gather quantitative data on the effectiveness of these measures. Machine learning algorithms offer an opportunity to infer subpopulations from complex SES data and help to identify high SES-risk subgroups. We hypothesized that clustering using SES risk factors would identify patient subpopulations within the CRC who are more likely to have more symptoms and worse clinical outcomes, and who are less likely to utilize CRC resources. This quantitative approach supports targeted interventions for patients most in-need of post-COVID care resources.

## Methods

### COVID Recovery center

The Brigham and Women’s Hospital (BWH) COVID-19 Recovery Center (CRC) was designed to incorporate strategies to address inequities in care. Our structured approach to comprehensive care in the CRC is particularly important for patients who are minorities, vulnerable, or disadvantaged. The CRC is a part of BWH (an academic medical center) and Brigham and Women’s Faulkner Hospital (an affiliated community hospital). Patients are referred through the hospital system (largely internally through primary care physicians or other clinicians in the system) or from the community. We began seeing patients in April 2021 and the effort is a multi-divisional collaboration, with subspecialty care including primary care, neurology, psychiatry, otolaryngology, cardiology, gastroenterology, rheumatology, allergy, dermatology, sleep medicine, physical medicine and partnerships with pharmacists and social workers. At the time of this study, the CRC had seen 1285 patients. Our core equity group designed strategies that promote equity through education and community partnerships, with a robust system of monitoring to ensure patients whom we serve in our recovery center reflect those who have borne a disproportionate burden of COVID-19. The multidisciplinary team also includes a community resource specialist who is an integral member in championing the equity mission within neighboring communities. To modify care barriers, we also reserved funds for transportation reimbursement, and created social support groups. This work was an iterative process, adapted throughout the year to focus on different aspects of community partnership building and use of tools that prioritized care for patients from communities most impacted. The CRC also provides opportunities to enroll eligible patients in the NIH Recover Study, supporting ongoing efforts to better understand post COVID impairments.

### Data collection: Variables, metrics and indicators for care delivery

Performance indicators designed prior to deployment of the CRC included indicators for completeness of data collection, proportional visits (patients seen in the CRC who were previously hospitalized, goal benchmark 30%), community engagement (patients referred to the CRC through community partnerships, goal benchmark at least 40%), interpreter access (percent of non-English speaking patients with timely access to interpreter services) and resource referral (percent of patients referred to social work or additional community resources). We prospectively collected data for research purposes as patients referred to the CRC. These data included demographic variables (including age, sex, race/ethnicity, sex); COVID-19 vaccination history, smoking, insurance status, COVID-19 related symptoms, support utilization, and additional variables described in detail below.

### Statistical analyses

#### Overview of study design

We included study participants with complete data for sex, race, language, and insurance status. We used these four socio-demographic variables as inputs into a clustering algorithm to classify individuals into subgroups, or clusters. We applied multivariable linear and logistic regression frameworks to test the association of clusters with anthropometric, clinical outcome, symptom, and resource utilization measures.

#### Cluster analyses

We used socio-demographic variables (sex, race, language, and insurance status (“government”, “commercial”, “other/none”)) to calculate Gower distances and create a distance matrix for non-continuous data. Gower distances provide a value between 0 and 1 that describes the dissimilarity of two data points; this approach, in contrast to Euclidean distance-based methods, allows one to quantify distances for mixed data types, including categorical, binary, and continuous variables [15, 16]. We then performed hierarchical clustering with complete linkage using the R cluster package. We chose these socio-demographic factors variables based on prior knowledge of SES risk factors (as described in the background [2, 5]) ease of variable acquisition, and completeness in the data. For specific variables, prior knowledge on the role as risk factors for acute infection or prolonged symptoms during COVID-recovery are described, such as for race [2], language/interpreter service use [17], sex [18], and insurance status [19]. To determine the optimal number of clusters, we computed the within-cluster sum-of-squares over a range of cluster numbers and chose the number of clusters at the inflection point at which additional clusters does not substantially improve error (i.e., inflection point on an “Elbow plot”). We assigned each patient a corresponding cluster number based on this hierarchical clustering algorithm.

#### Predictors

We coded cluster assignment as a categorical variable in which lowest ICU admissions cluster (cluster 2) was used as the reference group – that is, the group with the lowest initial disease severity.

#### Outcomes

We selected outcomes related to post-COVID-19 care based on clinician input. We examined the association of each predictor with the need for an ICU admission, symptoms, and utilization of SES-targeted resources. Symptoms included cough, dyspnea on exertion, anxiety, fatigue, and brain fog. SES-targeted resources and support utilization included social support services that provided one of the following services: psychoeducation for chronic illness, help navigating government benefits, help addressing financial/housing concerns and obtaining community resources, arranging for support groups, and providing care coordination.

#### Model specifications

We utilized multivariable linear and logistic regression models, as appropriate. We performed unadjusted and adjusted analyses. We adjusted all models for age in years, but not other socioeconomic factors as these variables were incorporated into the clustering analyses.

We performed all analyses in R v4.0.3 [20], a programming language and environment that supports data analyses and advanced graphical solutions. We performed linear regressions with the “lm” function and logistic regressions with the “glm” function. We assessed all variables for normality by visual inspection of histograms and Shapiro–Wilk tests. We compared continuous variables with Student t-tests or Wilcoxon tests, as appropriate. We compared categorical variables with analysis of variance (ANOVA) or Kruskal–Wallis tests, as appropriate. We reported 95% confidence intervals and considered two-sided *p*-values below 0.05 to be significant.

## Results

### Clustering analysis

**Fig. 1:**
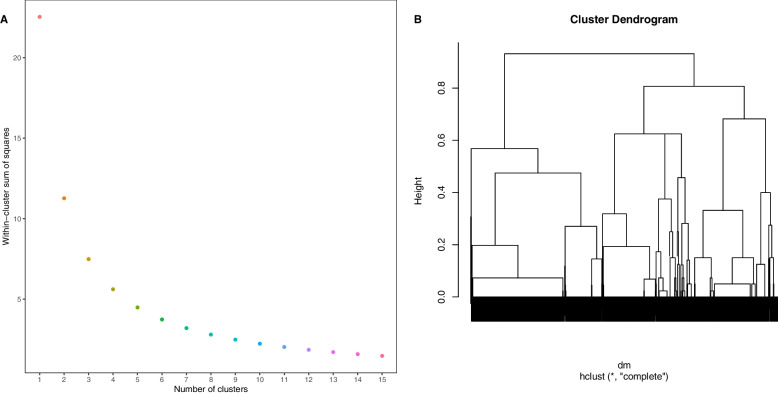
**A** Elbow plot showing the within-cluster sum of squares for varying numbers of clusters, **B** Dendogram of Cluster Analysis

We included 1285 patients with an average age of 47 years, predominantly female (71%) and White (78%). Previously hospitalized patients (average 15.9%) had a primary diagnosis of COVID-19. Discharge dates were not available but the average time from positive PCR to CRC appointment was 279 days. Using the socio-demographic factors of sex, race, language, and insurance status, we found that 3 clusters offered a marked reduction in the within-cluster sum of squares and that additional clusters did not substantially further reduce error (Fig. 1).

### Characteristics of cluster participants

We show the characteristics of patients within these clusters in Table 1. Cluster 2 patients are predominantly White with commercial insurance and had the lowest percentage of ICU admissions; this cluster was used as the reference group. Compared to cluster 2, Cluster 1 patients are more likely to be Latino/a, to utilize interpreter services, have government insurance, and to have had an ICU admission. Cluster 3 patients are more likely to be Black/African American or Latino/a and identify as non-Hispanic. Compared to cluster 2, cluster 3 patients are more likely to have had an ICU admission and utilize interpreter services, but also more likely to have commercial insurance.

**Table 1:** Cohort characteristics by cluster. Socio-demographic factors (sex, race, language, and insurance status (“government”, “commercial”, “other/none”)) were used to define patient clusters

**Characteristic**	**Cluster**			
	*1*	*2*	*3*	*P value*
n, total 1285	386	550	349	
Age in years (mean (SD))	47.24 (14.40)	47.86 (16.46)	46.95 (14.77)	0.738
Sex n, (%)				< 0.001
Male	38 (9.8)	47 (8.5)	280 (80.2)	
Female	343 (88.9)	501 (91.1)	69 (19.8)	
Nonbinary	5 (1.3)	2 (0.4)	0 (0.0)	
Race, n, (%)				< 0.001
Black/African American	26 (6.7)	0 (0.0)	71 (20.3)	
American Indian or Alaskan Native	1 (0.3)	0 (0.0)	1 (0.3)	
Asian	25 (6.5)	0 (0.0)	10 (2.9)	
Latino/a	88 (22.8)	1 (0.2)	44 (12.6)	
Other	2 (0.5)	11 (2.0)	8 (2.3)	
White	244 (63.2)	538 (97.8)	215 (61.6)	
Non-Hispanic Ethnicity n, (%)	291 (81.5)	373 (98.4)	214 (86.3)	< 0.001
Interpreter Utilization (%)	30 (78.9)	3 (2.9)	4 (8.3)	< 0.001
Smoking status, n, %				0.078
Current	11 (3.1)	20 (5.5)	11 (4.6)	
Former	66 (18.9)	88 (24.2)	41 (17.0)	
Never	273 (78.0)	256 (70.3)	189 (78.4)	
Insurance status, n, (%)				< 0.001
Commercial	244 (63.2)	387 (70.4)	309 (88.5)	
Government	142 (36.8)	157 (28.5)	40 (11.5)	
None	0 (0.0)	6 (1.1)	0 (0.0)	
ICU admission, n, (%)	16 (21.3)	22 (6.6)	37 (18.9)	< 0.001

*ICU* intensive care unit, *SD* standard deviation

### Clusters based on socio-demographic factors are associated with ICU admissions, symptoms, and resource utilization patterns

In Table 2, we show the age-adjusted odds ratios of selected outcomes by cluster. Compared to Cluster 2, we found that Cluster 1 patients are more likely to have required an ICU admission (OR 4.7 [95% CI: 2.1–10.6], *p* = 0.00023) and to report symptoms (ORs ranging 2.4–3.75), but less likely to utilize support for psychoeducation (OR 0.16 [95% CI: 0.07–0.4], *p* < 0.0001), support groups (OR 0.19 [95% CI: 0.07–0.56], *p* = 0.0026), and care coordination (OR 0.2 [95% CI: 0.06–0.69], *p* = 0.011). Cluster 3 patients demonstrate an increased odds for ICU admission (OR 3.15 [95% CI: 1.6–6.4], *p* = 0.0015), cough (OR 1.7 [95% CI: 1.1–2.6], *p* = 0.018), and dyspnea on exertion (OR 1.5 [95% CI: 1.02–2.3], *p* = 0.04); however, these patients utilized community resources at similar rates as cluster 2 patients, though there was a trend toward increased support for navigating government benefits.

**Table 2:** Age-adjusted logistic regression analyses of associations between cluster assignment and selected outcomes

Outcome	Age adjusted odds ratio, 95% CI; Cluster 1 Compared To Cluster 2	*P* value	Age adjusted odds ratio, 95% CI; Cluster 3 Compared To Cluster 2	*P* value
*ICU Admission*	4.67 (2.06—10.6)	0.00023	3.15 (1.55—6.39)	0.0015
*Symptom Report*				
Cough	3.75 (2.33—6.03)	< .0001	1.7 (1.1—2.64)	0.018
Dyspnea on exertion	3.35 (2.11—5.34)	< .0001	1.53 (1.02—2.3)	0.042
Anxiety	2.7 (1.58—4.63)	3E-04	0.858 (0.524—1.4)	0.54
Fatigue	3.26 (1.79—5.92)	1E-04	0.908 (0.57—1.45)	0.68
Brain fog	2.4 (1.42—4.05)	0.0011	0.826 (0.53—1.29)	0.4
*Type of Social Services Support*				
Psychoeducation for chronic illness	0.159 (0.0664—0.381)	< .0001	0.833 (0.477—1.46)	0.52
Navigating government benefits	1.1 (0.0686—17.7)	0.95	4.6 (0.475—44.5)	0.19
Financial/housing concerns	0.999 (0—Inf)	0.99	2.67e + 08 (0—Inf)	0.99
Support groups	0.191 (0.0652—0.56)	0.0026	0.546 (0.239—1.25)	0.15
Care coordination	0.198 (0.0571—0.685)	0.011	0.464 (0.168—1.28)	0.14
Provision of community resources	3.31e-08 (0—Inf)	0.99	1.22 (0.325—4.6)	0.77
Other	1.07 (0—Inf)	0.99	1.92e + 08 (0—Inf)	0.99

*ICU* intensive care unit

## Discussion

In this study of over 1,200 post-COVID patients recruited from a newly designed ambulatory COVID Recovery Center (CRC), we used four easily obtainable clinical variables (sex, self-reported race, language, insurance type) to identify three patient clusters with differences in the need for ICU admission, symptom reporting, and resource utilization. We found two clusters of predominantly non-White patients who were more likely to experience an ICU admission and report persistent symptoms, but one cluster who were less likely to utilize resources even after a CRC referral. These results highlight the need to improve not only access to multi-disciplinary specialty care, but also to address barriers to access within post-COVID care clinics.

We observed that the majority of CRC patients are English-speaking White women under 50 years of age with managed care, which highlights the need to improve CRC referral to expand inclusion of patients who demographically had higher rates of infection. Further, we identified one subset of patients (Cluster 1) who were primarily non-English-speaking, had government insurance, and a higher odds of ICU admission and symptoms, but who were less likely to access resources after CRC referral. We also identified a subset of primarily English-speaking and Black/African American patients (Cluster 3) with commercial insurance who had an increased odds of ICU admission but had similar resource utilization compared to our reference cluster. Taken together, these results suggest that 1) we need to improve referral volume of groups with a disproportionate burden of COVID infection, and 2) certain minority patients are not able to fully utilize the offered resources.

Our results demonstrating increased ICU admission in minority-predominant clusters are consistent with prior literature showing that minority populations share the disproportionate burden of severe disease [21]. A prior study of multihospital hospitalized patients in Michigan, combining clinical data with social vulnerability indices (SVI), found that patients from high SVI areas (who are more likely to be Black/African American or Hispanic) were more likely to have an ICU admission [10] and have acute organ dysfunction and failure. Both Cluster 1 and 3 patients experience greater ICU admissions and persistent symptoms. These associations may be explained by the fact that pulmonary sequala symptoms appear to be closely associated with acute infection severity [22], although the relationship may be nonlinear [23]. Understanding pulmonary impairments among racial/ethnic groups is also an area of ongoing research, with some data suggesting that Black adult patients hospitalized with COVID-19, at 6 months follow-up had lower percent predicted DLCO compared to Hispanic and White patients, after controlling for various predictors such as smoking status, ICU admission and history of chronic lung disease [24]. These data further support the need to purposefully engage and include minority patients in post-COVID evaluation and COVID recovery care.

We observed that patients referred to the CRC who were primarily non-English speaking individuals were less likely to utilize the offered resources, which suggests that post-COVID centers must be cognizant of within-center barriers to care. Recent data suggests that patients recovering from COVID-19 have increased risk of new onset mental health disorders, including anxiety [25]. It will be imperative to ensure that patients are comfortable reporting new mental health changes and to encourage resource utilization. The decreased utilization of psychoeducation and support groups is also notable because patients may experience mental health symptoms (Cluster 1 for example had increased odds of reporting anxiety) but be unlikely to seek specific support for a variety of reasons. The reason for this observation is unclear but may be attributable to cost of work-up and management/coverage concerns with reporting symptoms, language barriers, a paucity of Spanish-speaking healthcare providers, mistrust of social support systems, and stigma against psychoeducation. Other data during the pandemic also highlight the impact of broader societal events [26] on mental health. Improving access to interpreter services for healthcare providers, care coordination managers, social workers, and all other employees at the CRC and other similar centers will be critical to providing equitable care.

Despite an increase in ICU admissions and pulmonary symptoms, Cluster 3 patients reported similar non-pulmonary symptoms and appear effective at accessing support services, which may allude to multiple SES-related care barriers. There was a trend toward increased utilization of services for navigating government benefits; attempts to access support services suggests that these predominantly Black/African American and Latino/a patients may be living in areas with high social vulnerability index [10] and thus have a need for these specific resources. Whether Cluster 3 patients under-reported or did not experience non-pulmonary post-COVID-19 symptoms (e.g. anxiety) is unclear. Symptom underreporting amongst minority communities is well-described in oncological literature and is attributed to a variety of individual and societal factors [27]. Additionally, patients may not report symptoms during initial intake which is frequently remote (via electronic health record or telephone), therefore this divide may modify both report and capture of this information. This barrier to care may be present across many post-COVID care centers. The optimal strategies to mitigate underreporting are unclear, but include standardized approaches to symptom monitoring, actively offering resources and providing extra assistance in accessing resources, recruitment and retention of healthcare providers from minority communities, and building long-term trustworthy partnerships within the community. Lastly, more granular documentation of request and provision of services would be helpful to better monitor needs. Ensuring minority patients have community mental health resources will be important to support their recovery. Reducing housing insecurity and supporting affordable housing in the context of COVID-19 and beyond also presents an opportunity for focused discussion and policies. Further investigation into the socioeconomic factors affecting this patient subgroup is needed.

Strengths of this study include a relatively large, well-characterized sample of post-COVID patients in whom data were collected prospectively for research purposes and the subsequent application of a clustering algorithm to identify patient subgroups. To our knowledge, this is the first study of outpatient COVID recovery care to apply quantitative machine-learning methods to understand differential resource utilization and symptom reporting within a recovery center. Such a clustering method can be used to categorize individuals into high-risk groups in other populations. As more data analytics emerge to identify patients who may have post-COVID symptoms [28] for a variety of clinical and research purposes, ensuring equity will be of high importance. Limitations of this study include that, by design, it is a single cohort study of an ongoing project. Replication in other centers is needed as well as the development and validation of SES-risk prediction tools. We were able to identify specific care barriers (i.e... access to interpreter services and within center resource utilization), but we have yet to measure the effects of interventions targeting these factors. Future studies can address these issues.

## Conclusions

In conclusion, we used a clustering algorithm to define patient subpopulations using sex, race, language, and insurance status in a post-COVID clinic, and demonstrated a high-risk subgroup who had more ICU admissions and more symptoms, but less resource utilization. Overall, these data allow us to consider patient-centered approaches for individual patients and subpopulations of patients with shared needs. Future studies will focus on targeted approaches to promote resource utilization which may ultimately improve individual outcomes and provide a framework to improve the equity of care across the hospital system.

## Supplementary Information

**Additional file 1.** 

## Data Availability

Data is not available to share publicly.

## Abbreviations

ANOVA: Analysis of variance
BWH: Brigham and Women’s Hospital
COVID-19: Coronavirus disease
CRC: COVID Recovery Center
ICU: Intensive Care Unit
SES: Socioeconomic status
SD: Standard deviation

## References

[1] Okonkwo Nneoma E (2020). COVID-19 and the US response: accelerating health inequities. BMJ Evid Based Med.

[2] Hooper MW, Napoles AM, Perez-Stable EJ. COVID-19 and racial/ethnic disparities. JAMA. Published online May 11, 2020.

[3] Elo IT, Luck A, Stokes AC, Hempstead K, Xie W, Preston SH (2022). Evaluation of age patterns of COVID-19 mortality by race and ethnicity from March 2020 to October 2021 in the US. JAMA Netw Open.

[4] “COVID-19 Weekly Cases and Deaths per 100,000 Population by Age, Race/Ethnicity, and Sex”. Centers for Disease Control and Prevention. Available from: https://covid.cdc.gov/covid-data-tracker/#demographicsovertime. Accessed 19 Sept 2022.

[5] Li J, Wang X, Yuan B (2022). Population distribution by ethnicities and the disparities in health risk and coping in the United States during the pandemic: the spatial and time dynamics. Arch Public Health.

[6] Berkowitz, Rachel L., et al. "Structurally vulnerable neighborhood environments and racial/ethnic COVID-19 inequities." Cities Health (2020): 1–4. 10.1080/23748834.2020.1792069 PMC9216191 35747269

[7] Williams DR, Cooper LA. COVID-19 and health equity – a new kind of “Herd Immunity”. JAMA. Published online May 11, 2020. 10.1001/jama.2020.8051 32391852

[8] Lane Fall MB, Iwashyna TJ, Cooke CR, Benson NM, Kahn JM (2012). Insurance and racial differences in long-term acute care utilization after critical illness. Crit Care Med.

[9] Jolley S, Nordon-Craft A, Wilson MP (2022). Disparities in the allocation of inpatient physical and occupational therapy services for patients with COVID-19. J Hosp Med.

[10] Tipirneni R, Karmakar M, O'Malley M, Prescott HC, Chopra V (2022). Contribution of individual- and neighborhood-level social, demographic, and health factors to COVID-19 hospitalization outcomes. Ann Intern Med.

[11] Tukpah AM, Moll M, Gay E (2021). COVID-19 racial and ethnic inequities in acute care and critical illness survivorship. Ann Am Thorac Soc.

[12] Santhosh L, Block B, Kim SY, Raju S, Shah RJ, Thakur N, Brigham EP, Parker AM (2021). Rapid design and implementation of Post-COVID-19 clinics. Chest.

[13] Corbie-Smith G, Wolfe MK, Hoover SM, Dave G (2021). Centering equity and community in the recovery of the COVID-19 Pandemic. N C Med J.

[14] Tangcharoensathien V, Carroll D, Lekagul A (2022). Resilient and equitable recovery from the covid-19 pandemic. BMJ.

[15] Gower JC (1971). A general coefficient of similarity and some of its properties. Biometrics.

[16] Gower JC, Legendre P (1986). Metric and Euclidean properties of dissimilarity coefficients. J Classif.

[17] Cohen-Cline H, Li HF, Gill M (2021). Major disparities in COVID-19 test positivity for patients with non-English preferred language even after accounting for race and social factors in the United States in 2020. BMC Public Health.

[18] Fernández-de-Las-Peñas C, Martín-Guerrero JD, Pellicer-Valero ÓJ, Navarro-Pardo E, Gómez-Mayordomo V, Cuadrado ML, Arias-Navalón JA, Cigarán-Méndez M, Hernández-Barrera V, Arendt-Nielsen L (2022). Female sex is a risk factor associated with long-term Post-COVID related-symptoms but not with COVID-19 symptoms: the LONG-COVID-EXP-CM multicenter study. J Clin Med.

[19] McCain JL, Wang X, Connell K, Morgan J (2022). Assessing the impact of insurance type on COVID-19 mortality in black and white patients in the largest healthcare system in the state of georgia. J Natl Med Assoc.

[20] “What is R?” The R Foundation. Available from: https://www.r-project.org/about.html. Accessed 10 Jan 2023.

[21] Magesh S, John D, Li WT, Li Y, Mattingly-App A, Jain S, Chang EY, Ongkeko WM (2021). Disparities in COVID-19 outcomes by race, ethnicity, and socioeconomic status: a systematic-review and meta-analysis. JAMA Netw Open.

[22] Jiang DH, Roy DJ, Gu BJ, Hassett LC, McCoy RG (2021). Postacute sequelae of severe acute respiratory syndrome Coronavirus 2 infection: a State-of-the-art review. JACC Basic Transl Sci.

[23] Boutou AK, Asimakos A, Kortianou E, Vogiatzis I, Tzouvelekis A (2021). Long COVID-19 Pulmonary Sequelae and Management Considerations. J Pers Med.

[24] Konkol SB, Ramani C, Martin DN, Harnish-Cruz CK, Mietla KM, Sessums RF, Widere JC, Kadl A (2022). Differences in lung function between major race/ethnicity groups following hospitalization with COVID-19. Respir Med.

[25] Xie Y, Xu E, Al-Aly Z (2022). Risks of mental health outcomes in people with covid-19: cohort study. BMJ.

[26] Thomeer MB, Moody MD, Yahirun J (2022). Racial and ethnic disparities in mental health and mental health care during the COVID-19 Pandemic. J Racial Ethn Health Disparities.

[27] Bulls HW, Chang PH, Brownstein NC, Zhou JM, Hoogland AI, Gonzalez BD, Johnstone P, Jim HSL (2022). Patient-reported symptom burden in routine oncology care: examining racial and ethnic disparities. Cancer Rep (Hoboken).

[28] Pfaff  ER, Girvin AT, Bennett  TD, Bhatia  A, Brooks  IM, Deer  RR, Dekermanjian  JP,  Jolley  SE, Kahn  MG, Kostka  K, McMurry  JA, Moffitt  R, Walden  A, Chute  CG, Haendel  MA (2022). N3C Consortium. Identifying who has long COVID in the USA: a machine learning approach using N3C data. Lancet Digit Health.

