# Supplementary material for: Cluster analysis of COVID-19 recovery center patients at a clinic in Boston, MA 2021–2022: impact on strategies for access and personalized care
Source: Arch Public Health. 2023 Mar 14;81:39. doi: 10.1186/s13690-023-01033-2 (PMC10011754; doi:10.1186/s13690-023-01033-2)
Supplement: Supplementary file 1 — Additional file 1. [file 13690_2023_1033_MOESM1_ESM.docx]

**Aims and Scope statement**

1. What is known?

The COVID-19 pandemic disproportionately impacted historically disadvantaged communities of color and patients from socioeconomically under-resourced backgrounds. Prior to the pandemic, data suggested that there were differential rates in access to outpatient subspecialty care and long-term acute care for non-White patients. Given the burden of the pandemic and pre-existing challenges, we anticipated that access to outpatient COVID-19 recovery services would be limited for minority patients, an inequity which would constrain both high-quality clinical care and reduce the maximal benefit of public health interventions. Despite the expansion of COVID-related research, there remains a dearth of quantitative data analyses describing patient reported symptoms/experiences during the COVID-recovery period and the facilitators or barriers to creation of necessary support mechanisms.

1. What does the study add?

To our knowledge, this is the first study of outpatient COVID recovery care to apply quantitative machine-learning methods to understand differential resource utilization and symptom reporting within a recovery center. This data helps describe the relationships between COVID-19 infection rates, hospitalizations and recovery care in subpopulations of patients. Through analysis of data collected from our 1285 recovery center patients, we discovered subpopulations of patients with different patterns of care utilization. Patients in a cluster of largely Latino/a ethnicity, on government insurance and requiring interpreter service utilization, were statistically significantly more likely to report all five symptoms assessed, especially fatigue, cough, and dyspnea on exertion; they also had 3.8 times increase in the odds of an ICU admission. Additionally, a cluster of patients more likely to be Black/African American or Latino/a, identify as non-Hispanic and to not utilize interpreter services, were more likely to seek specific services including government benefits and community resources. They were also less likely to report symptoms. Knowledge garnered through this data analysis can guide anticipation of possible barriers to symptom reporting and resource delivery and promote optimal personalized care to all patients regardless of socioeconomic status.

1. What are implications for clinical practice, public health and / or research?

This data provides insights into ambulatory COVID-19 recovery patient populations. These insights from different subpopulations of patients can inform targeted strategies which are tailored to specific patient needs. By recognizing the impact of social determinants of health on both acute infection and recovery, we can prioritize the needs of patients from communities most impacted by COVID. Disseminating this research can support clinicians, researchers, and policy makers in designing tools that incorporate unique attributes of individual patients or subpopulations of patients, thereby promoting investments in patient-centered, equitable approaches to high-value care.
